# Possible role of *TLR4* and *TLR9* SNPs in protection against congenital toxoplasmosis

**DOI:** 10.1007/s10096-015-2461-3

**Published:** 2015-08-09

**Authors:** W. Wujcicka, Z. Gaj, J. Wilczyński, D. Nowakowska

**Affiliations:** Scientific Laboratory of the Center of Medical Laboratory Diagnostics, Polish Mother’s Memorial Hospital—Research Institute, 281/289 Rzgowska Street, Lodz, 93-338 Poland; Department of Perinatology and Gynecology, Polish Mother’s Memorial Hospital—Research Institute, Lodz, Poland

## Abstract

The purpose of this investigation was the determination of the distribution of genotypes at single nucleotide polymorphisms (SNPs) of the toll-like receptor 4 (*TLR4*) and the toll-like receptor 9 (*TLR9*) in fetuses and newborns congenitally infected with *Toxoplasma gondii* and the identification of genetic changes predisposing to infection development. The study involved 20 fetuses and newborns with congenital toxoplasmosis and 50 uninfected controls. The levels of IgG and IgM antibodies against *T. gondii*, as well as IgG avidity, were estimated by enzyme-linked fluorescent assay (ELFA) tests. *T. gondii* DNA loads in amniotic fluids were assayed by the real-time (RT) quantitative polymerase chain reaction (Q PCR) technique for parasitic *B1* gene. *TLR4* and *TLR9* SNPs were identified using a self-designed multiplex nested PCR-restriction fragment length polymorphism (RFLP) assay. Randomly selected genotypes at SNPs were confirmed by sequencing. All the genotypes were tested for Hardy–Weinberg equilibrium and *TLR4* genotypes were analyzed for linkage disequilibrium. A correlation was studied between the genotypes or haplotypes and the development of congenital toxoplasmosis using a logistic regression model. Single SNP analysis showed no statistically significant differences in the distribution of distinct genotypes at the analyzed *TLR4* and *TLR9* SNPs between *T. gondii*-infected fetuses and newborns and the controls. Taking into account the prevalence of alleles residing within polymorphic sites, similar prevalence rates were observed in both of the studied groups. The multiple SNP analysis indicated GTG variants at the *TLR4* and *TLR9* SNPs to be significantly less frequent in offspring with congenital toxoplasmosis than in uninfected offspring (*p* ≤ 0.0001). *TLR4* and *TLR9* SNPs seem to be involved in protection against congenital toxoplasmosis.

## Introduction

Intrauterine infections are among the major causes of perinatal morbidity and mortality. Nearly 40 % of pregnant Polish women are infected with *Toxoplasma gondii* [1–3]. Particularly dangerous are primary infections in pregnant women, which are usually asymptomatic and, in approximately 30–50 % of patients, result in transplacental transmission of *T. gondii* to the fetus [4]. In fetuses and newborns with immature immune systems, consequential congenital infections may bring about a very severe, if not fatal, course [4, 5]. Congenital toxoplasmosis occurs in approximately 0.07–2.9 % of live births.

Toll-like receptor (TLR)/MyD88 signaling has been reported as the key pathway in a non-specific antimicrobial response against *T. gondii* [6, 7]. The glycosylphosphatidylinositol (GPI) of *T. gondii* was demonstrated to trigger *TLR4* signaling pathways [8–10]. In inflammatory monocytes, *T. gondii* infection induced the production of interferon (IFN)-β through *TLR4* and MyD88 signaling [11]. A lack of tumor necrosis factor (TNF) expression after *T. gondii* infection was characteristic for mice deficient in *TLR2* and *TLR4*, but not in *TLR2* molecules alone [8]. Also, *TLR9* might have an important role in the immunity against *T. gondii*, since *TLR9*-deficient mice, infected with the parasite, were comparatively resistant to the ileitis, revealing a decreased Th1 immune response [12, 13]. Taking into account *TLR* genetic changes, possibly involved in the development of *T. gondii* infections, the *TLR9* 1635 A>G single nucleotide polymorphism (SNP) was reported to be associated with toxoplasmic retinochoroiditis among Brazilian children with ocular toxoplasmosis [14]. So far, no paper has ever reported the role of *TLR4* and *TLR9* SNPs in the development of congenital infections with *T. gondii*. However, previous studies commonly showed the role of *TLR4* 896 A>G and 1196 C>T SNPs in several pregnancy disorders, including premature rupture of membranes (PROM), bronchopulmonary dysplasia, and preterm labor [6, 15, 16]. In turn, the *TLR9* 1635 A>G SNP was also reported to be involved in cervical cancer, mother-to-child transmission (MTCT) of human immunodeficiency virus type 1 (HIV-1), and an increased risk of low birth weight in infants, a risk of maternal anemia, as well as a clinical picture of malaria in pregnancy [17–19].

Considering the role of TLR molecules in the development of *T. gondii* infection, as well as *TLR* genetic changes in pregnancy disorders, we decided to describe in this report the prevalence rates of the genotypes and alleles at the *TLR4* 896 A>G, 1196 C>T, and *TLR9* 1635 G>A SNPs in fetuses and newborns congenitally infected with *T. gondii* and compare them to the prevalence rates observed in uninfected controls. The haplotype prevalence rates were analyzed for *TLR4* SNPs as well. Moreover, a multiple SNP analysis was performed to identify the combined *TLR* SNP variants possibly involved in the immune response against *T. gondii*. Additionally, we also determined genotypes and alleles at the *TLR2* 2258 G>A SNP (rs5743708) among the studied groups of patients. However, in the case of the *TLR2* SNP, no contribution was found to congenital infection with *T. gondii*; hence, no molecular data on this polymorphism were shown in the current study.

## Materials and methods

The study was performed with 20 fetuses and newborns with congenital toxoplasmosis and 50 control fetuses and newborns without *T. gondii* intrauterine infection. Samples were collected both retrospectively (15 infected cases and 23 controls) and prospectively (five infected cases and 27 controls). The specimens, selected for genetic studies of the infected patients, included fetal amniotic fluid samples, obtained via amniocentesis in pregnant women, all of them treated at the Polish Mother’s Memorial Hospital Research Institute in Lodz, between the years 2000 and 2014. The diagnosis of intrauterine infections was based on maternal serological tests and the clinical picture, including flu-like symptoms, fetal and neonatal ultrasound markers related to the diseases, and the presence of parasite or viral DNA in body fluids of the patients. Congenital infections with *T. gondii* were confirmed by the presence of pathogen DNA in body fluids (amniotic and/or ascitic fluids, umbilical cord blood) of the fetuses and newborns. The group of control cases included the offspring of pregnant women seronegative for *T. gondii*. The study was approved by the Research Ethics Committee at the Polish Mother’s Memorial Hospital Research Institute. The samples, previously collected for diagnostic purposes and anonymized, were used for molecular analysis. Informed consent forms were signed by the enrolled pregnant women prior to the study.

### Serological tests

Blood specimens were collected from the pregnant women participating in the study by venipuncture on the first visit to the Institute. Serum samples were obtained by centrifugation and then stored at 4 °C before analysis. Serological tests were performed at the Department of Clinical Microbiology of the Institute.

Screening for *T. gondii* IgG antibodies was performed with the enzyme-linked fluorescent assay (ELFA) VIDAS TOXO IgG II (bioMérieux), while the levels of IgM antibodies were estimated with the ELFA assay VIDAS TOXO IgM (bioMérieux). *T. gondii* IgG avidity was determined using the ELFA assay VIDAS TOXO IgG Avidity (bioMérieux). The pregnant women were determined as possibly infected with *T. gondii* in case of seroconversion during pregnancy and/or serology, suggesting recent infection, based on IgM seropositivity, as well as on low IgG avidity index. In order to confirm recent infection, the suspected women, as well as their fetuses and newborns, were tested for the presence of parasite DNA, using real-time (RT) quantitative polymerase chain reaction (Q PCR) assays for *T. gondii B1* gene fragments.

### DNA isolation

Genomic DNA was extracted from 5 ml of the amniotic fluid, from 3 ml of the cerebrospinal fluid, or from 200 μl of the umbilical cord blood of the fetuses, using a High Pure PCR Template Preparation Kit (Roche, Mannheim, Germany), and submitted to genetic studies of congenital toxoplasmosis. The extracted DNA was diluted in 100 μl of elution buffer and stored at −20 °C until molecular analyses.

### Detection and quantification of *T. gondii* DNA

Parasite DNA was identified and quantified by a RT Q PCR assay for a *B1* gene fragment of 83 bps in length, as previously described [20]. Forward and reverse primers and TaqMan MGB probe sequences were as follows: 5′-CAAGCAGCGTATTGTCGAGTAGAT-3′, 5′-GCGTCTCTTTCATTCCCACATTTT-3′, and 5′-6-FAM-CAGAAAGGAACTGCATCCGTT-NFQ-3′, respectively [20]. Standard curves, used in the quantitative analyses, were plotted with serial 10-fold dilutions from 10^5^ to 1 copy of *T. gondii* RH strain (tebu-bio; 08-948-250). *T. gondii* DNA copy numbers, present in PCR samples, were determined by the absolute quantification analysis, done automatically by the software, applied with the RT PCR instrument. RT Q PCR assays were performed using a LightCycler® 2.0 Instrument (Roche, Mannheim, Germany).

### Determination of SNPs located in *TLR4* and *TLR9* genes

**Fig. 1:**
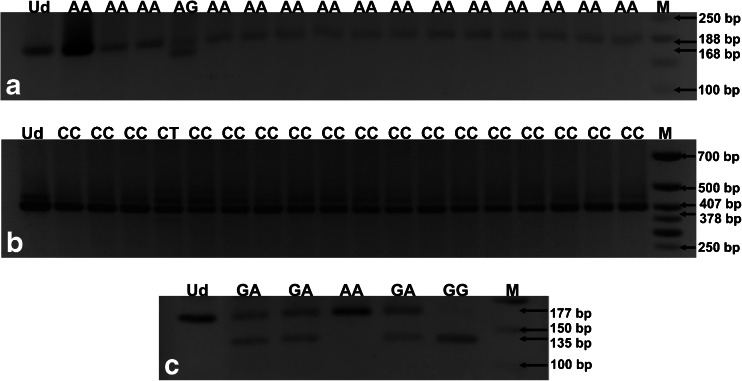
Agarose gel electrophoresis of polymerase chain reaction-restriction fragment length polymorphism (PCR-RFLP) products for profiling genotypes at the toll-like receptor 4 (*TLR4*) 896 A>G single-nucleotide polymorphism (SNP) (**a**), the *TLR4* 1196 C>T SNP (**b**), and the *TLR9* 1635 G>A SNP (**c**). DNA fragments which resulted from the restriction analyses performed with NcoI (**a**), HinfI (**b**), and BstUI (**c**) endonucleases were separated in 2 % agarose gels and stained with ethidium bromide. The numbers on the right-hand side show the size of resolved DNA fragments. *M* 50-bp DNA marker; *Ud* undigested PCR product; *AA*, *AG*, *GG*, *GA*, *CC*, *CT*, *TT* genotypes at the studied *TLR* polymorphisms

**Fig. 2:**
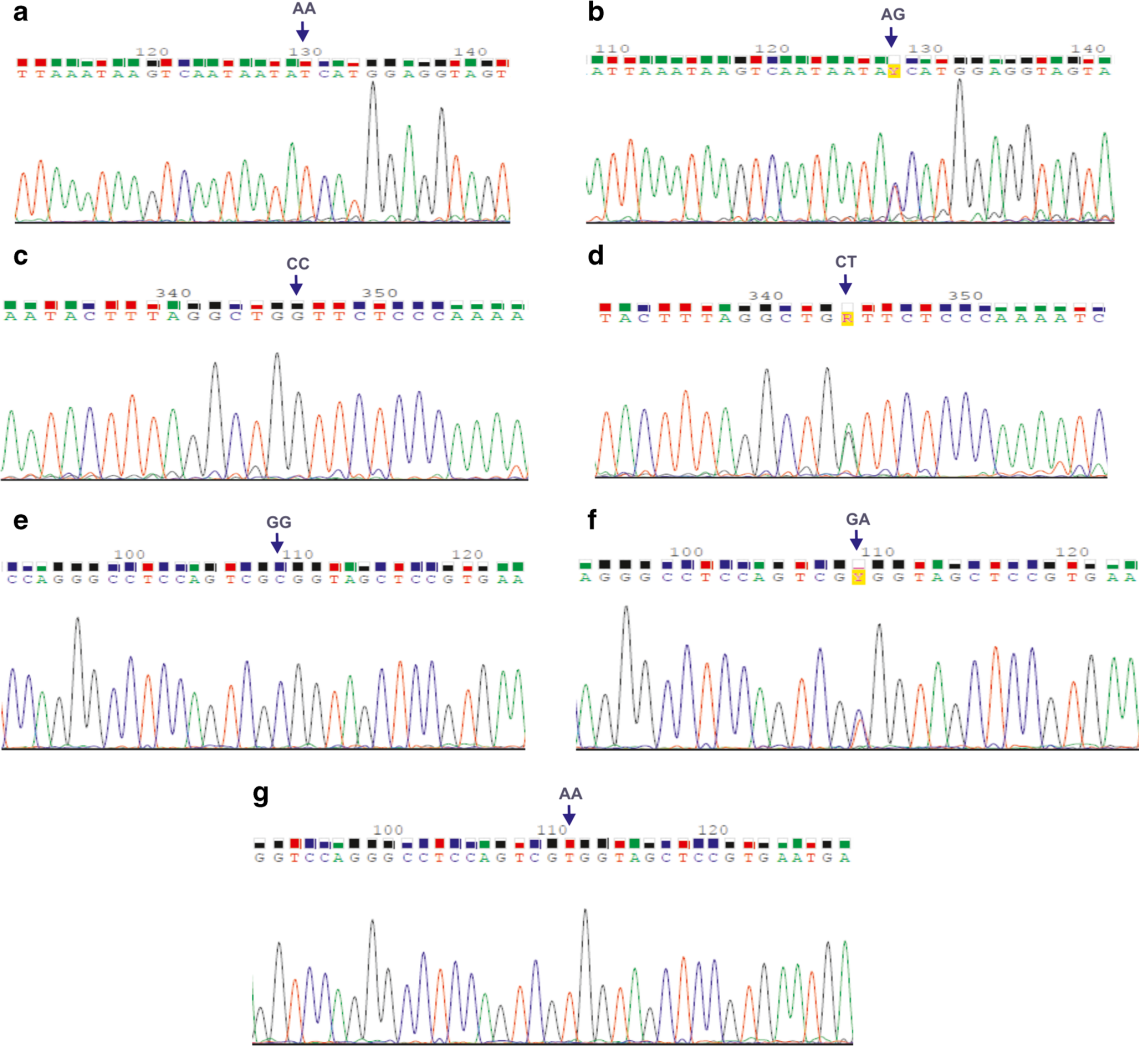
Chromatograms of DNA sequences containing the *TLR4* 896 A>G SNP (**a**, **b**), the *TLR4* 1196 C>T SNP (**c**, **d**), and the *TLR9* 1635 G>A SNP (**e**–**g**). DNA reverse strands were sequenced for all the analyzed SNPs. *AA*, *AG*, *CC*, *CT*, *GG*, *GA* genotypes at the described *TLR* SNPs

The *TLR4* 896 A>G and 1196 C>T SNPs and the *TLR9* 1635 G>A SNP were determined using the self-designed multiplex nested PCR assay described in our recent paper [21]. The sequences of external and internal primers, amplicon lengths, and annealing temperatures used in the multiplex PCR are shown in Table 1. External primer sequences were designed using the Vector NTI Suite 5.5 software, whereas the internal primers were taken from the literature [22–24]. Multiplex nested PCR products were digested with NcoI, HinfI, or BstuI endonucleases to determine the genotypes at the *TLR4* 896 A>G, *TLR4* 1196 C>T, or *TLR9* 1635 G>A SNPs, respectively. *TLR* SNPs and genotypes were discriminated based on the length of restriction fragments ([22–24]; see Table 1, Fig. 1). Genotypes at the *TLR4* 896 A>G SNP were assessed for 69 out of the 70 studied fetuses and neonates, at the *TLR4* 1196 C>T SNP for 59 out of the 70 patients, while the genetic variants at the *TLR9* 1635 G>A SNP were assessed for 64 out of the 70 analyzed offspring. Randomly selected PCR products for the *TLR4* and *TLR9* SNP genotypes were then sequenced by the Sanger method to confirm the outcomes of the multiplex nested PCR-restriction fragment length polymorphism (RFLP) assay. In case of *TLR4* SNPs, the sequencing was performed for three AA homozygotes and one AG heterozygote at the *TLR4* 896 A>G SNP, as well as for two CC homozygotes and one CT heterozygote at the *TLR4* 1196 C>T SNP. Considering the *TLR9* SNP, the sequencing was performed for four GG homozygotes, ten GA heterozygotes, and three AA homozygotes. The chromatograms, which illustrate the DNA sequences of PCR products for different *TLR* SNPs, are shown in Fig. 2. Both the sequenced and the reference fragments of the analyzed *TLR* genes were compared, using the BLASTN program for alignment of two (or more) sequences (http://blast.ncbi.nlm.nih.gov/Blast.cgi?PAGE_TYPE=BlastSearch&BLAST_SPEC=blast2seq&LINK_LOC=align2seq), and chromatograms were read using the Sequence Scanner 1.0 and the Chromas Lite 2.1.1 programs.

**Table 1:** Primers, annealing temperatures, and the lengths of products obtained in the multiplex nested polymerase chain reaction (PCR) assay for single nucleotide polymorphisms (SNPs) in the toll-like receptor 4 (*TLR4*) and toll-like receptor 9 (*TLR9*) genes

Gene	SNP^a^ name	Primer sequences (5′-3′)		Annealing temperature (°C)	Amplicon length (bps)^b^	Restriction enzyme	Profile (bps)
*TLR4*	896 A>G (1063 A>G, rs4986790)	External	For: AAAACTTGTATTCAAGGTCTGGC	52	355	NcoI	AA: 188 AG: 188, 168, 20 GG: 168, 20
			Rev: TGTTGGAAGTGAAAGTAAGCCT				
		Internal	For: AGCATACTTAGACTACTACCTCCATG	61	188		
			Rev: AGAAGATTTGAGTTTCAATGTGGG				
	1196 C>T (1363 C>T, rs4986791)	External	For: AGTTGATCTACCAAGCCTTGAGT	52	510	HinfI	CC: 407 CT: 407, 378, 29 TT: 378, 29
			Rev: GGAAACGTATCCAATGAAAAGA				
		Internal	For: GGTTGCTGTTCTCAAAGTGATTTTGGGAGAA	59	407		
			Rev: ACCTGAAGACTGGAGAGTGAGTTAAATGCT				
*TLR9*	1635 G>A (2848G>A, rs352140)	External	For: GTCAATGGCTCCCAGTTCC	52	292	BstUI	GG: 135, 42 GA: 177, 135, 42 AA: 177
			Rev: CATTGCCGCTGAAGTCCA				
		Internal	For: AAGCTGGACCTCTACCACGA	59	177		
			Rev: TTGGCTGTGGATGTTGTT				

^a^ *SNP* single nucleotide polymorphism

^b^ *bps* base pairs

### Statistical analysis

The prevalence rates of genotypes and alleles at the *TLR4* and *TLR9* SNPs that were identified in the examined fetuses and newborns, both those infected with *T. gondii* and the controls, were estimated by means of descriptive statistics. The study groups were tested for the Hardy–Weinberg (H-W) equilibrium, linkage disequilibrium (LD), and haplotypes using the SNPStats software (http://bioinfo.iconcologia.net/en/SNPStats_web). The relationship between the genotypes or alleles at the *TLR* SNPs and the development of *T. gondii* infection was determined using cross-tabulation and Pearson’s Chi-squared test, as well as the logistic regression model. The estimation of haplotypes at the *TLR4* SNPs, as well as the multiple SNP analysis, was performed employing the expectation–maximization (EM) algorithm. All the results were determined to be statistically significant when they reached the significance level of *p* ≤ 0.050. The statistical analysis was, in part, supported by the NCSS 97 software.

## Results

### *T. gondii* DNA loads in fetal and neonatal body fluids

The median load of *T. gondii* DNA in the collected fetal amniotic fluid samples was 6.5 × 10^2^ copies/ml and ranged from 3.0 to 9.7 × 10^3^ copies/ml, while the mean parasitic load was 2.0 × 10^3^ copies/ml. In case of cerebrospinal fluid samples, the median *T. gondii* DNA load was 1.5 × 10^2^ copies/ml, ranging from 1.1 × 10 to 1.2 × 10^5^ copies/ml, and the mean parasitic load was 3.0 × 10^4^ copies/ml. In neonatal umbilical cord blood specimens, the parasitic load was 7.5 × 10^3^ copies/ml.

### Hardy–Weinberg equilibrium, linkage disequilibrium

The genotypes at the *TLR4* 896 A>G SNP preserved the H-W equilibrium in *T. gondii*-infected fetuses and newborns (*p* = 1.000), while they were not in the H-W equilibrium among the uninfected cases (*p* ≤ 0.050). The genotypes at the *TLR4* 1196 C>T SNP did not preserve the H-W equilibrium in either of the analyzed groups (*p* ≤ 0.050). In turn, the prevalence rates of genotypes at the analyzed *TLR9* 1635 G>A SNP were in the H-W equilibrium, both in the infected subjects as well as in uninfected controls (*p* = 1.000). The *TLR4* 896 A>G and 1196 C>T SNPs were seen in linkage disequilibrium among both the infected and the control offspring (*p* ≤ 0.050).

### Prevalence rates of the genotypes at the *TLR4* 896 A>G, 1196 C>T, and *TLR9* 1635 G>A SNPs

Among the fetuses and newborns infected with *T. gondii*, the prevalence rates of the AA and AG genotypes at the *TLR4* 896 A>G SNP were 94.7 % (18/19) and 5.3 % (1/19), respectively (see Table 2). In case of the *TLR4* 1196 C>T SNP, the CC and CT genotypes were observed in 94.4 % (17/18) and 5.6 % (1/18) of the patients, respectively. For the *TLR9* 1635 G>A SNP, the GG, GA, and AA genotypes occurred in 20.0 % (4/20), 55.0 % (11/20), and 25.0 % (5/20) of the fetuses and newborns, respectively. Among the uninfected patients, the prevalence rates of the AA, AG, and GG genotypes at the *TLR4* 896 A>G SNP were 94.0 % (47/50), 2.0 % (1/50), and 4.0 % (2/50), respectively. The CC, CT, and TT genotypes at the *TLR4* 1196 C>T SNP were carried by 87.8 % (36/41), 7.3 % (3/41), and 4.9 % (2/41) of cases, respectively. Regarding the *TLR9* SNP, the prevalence rates of the GG, GA, and AA genotypes were 18.2 % (8/44), 47.7 % (21/44), and 34.1 % (15/44), respectively. No relationship was observed between single genotypes at the studied *TLR* SNPs and congenital toxoplasmosis development. Taking into account all the analyzed SNPs, the multiple ACG variant at the *TLR4* 896 A>G, 1196 C>T, and *TLR9* 1635 G>A polymorphic sites was most common among the fetuses and newborns with congenital toxoplasmosis, with a prevalence rate of 52.8 %. In the group of congenitally infected offspring, the ACA and GTA variants were less frequently observed (44.5 % and 2.8 %, respectively). Considering the uninfected controls, the most common multiple variant was ACA (56.6 %), while the ACG, GTA, ATG, and GTG variants were less frequently observed (34.9 %, 4.4 %, 2.4 %, and 1.7 %, respectively). The GTG variants were significantly less frequent among the fetuses and newborns with congenital toxoplasmosis as compared to the uninfected offspring [0.0 % vs. 1.7 %, respectively; odds ratio (OR) 6.0 × 10^7^; *p* ≤ 0.0001].

**Table 2:** Single SNP analysis of the relationship between *TLR* polymorphisms and congenital *Toxoplasma gondii* infection

Gene polymorphism	Genetic model	Genotype	Genotype prevalence rates; *n* (%)^a^		OR^b^ (95 % CI)^c^	*p*-Value^d^
			Infected cases	Uninfected controls		
*TLR4* 896 A>G	Codominant	AA	18 (94.7 %)	47 (94 %)	1.00	0.420
		AG	1 (5.3 %)	1 (2 %)	2.61 (0.15–44.01)	
		GG	0 (0 %)	2 (4 %)	0.00 (0.00–NA)	
	Dominant	AA	18 (94.7 %)	47 (94 %)	1.00	0.910
		AG-GG	1 (5.3 %)	3 (6 %)	0.87 (0.08–8.92)	
	Recessive	AA-AG	19 (100 %)	48 (96 %)	1.00	0.250
		GG	0 (0 %)	2 (4 %)	0.00 (0.00–NA)	
	Overdominant	AA-GG	18 (94.7 %)	49 (98 %)	1.00	0.490
		AG	1 (5.3 %)	1 (2 %)	2.72 (0.16–45.85)	
	Log-additive	–	–	–	0.66 (0.12–3.75)	0.620
*TLR4* 1196 C>T	Codominant	CC	17 (94.4 %)	36 (87.8 %)	1.00	0.450
		CT	1 (5.6 %)	3 (7.3 %)	0.71 (0.07–7.3)	
		TT	0 (0 %)	2 (4.9 %)	0.00 (0.00–NA)	
	Dominant	CC	17 (94.4 %)	36 (87.8 %)	1.00	0.410
		CT-TT	1 (5.6 %)	5 (12.2 %)	0.42 (0.05–3.91)	
	Recessive	CC-CT	18 (100 %)	39 (95.1 %)	1.00	0.220
		TT	0 (0 %)	2 (4.9 %)	0.00 (0.00–NA)	
	Overdominant	CC-TT	17 (94.4 %)	38 (92.7 %)	1.00	0.800
		CT	1 (5.6 %)	3 (7.3 %)	0.75 (0.07–7.69)	
	Log-additive	–	–	–	0.43 (0.07–2.78)	0.300
*TLR9* 1635 G>A	Codominant	AA	5 (25 %)	15 (34.1 %)	1.00	0.760
		GA	11 (55 %)	21 (47.7 %)	1.57 (0.45–5.47)	
		GG	4 (20 %)	8 (18.2 %)	1.50 (0.31–7.21)	
	Dominant	AA	5 (25 %)	15 (34.1 %)	1.00	0.460
		GA-GG	15 (75 %)	29 (65.9 %)	1.55 (0.47–5.09)	
	Recessive	AA-GA	16 (80 %)	36 (81.8 %)	1.00	0.860
		GG	4 (20 %)	8 (18.2 %)	1.12 (0.3–4.28)	
	Overdominant	AA-GG	9 (45 %)	23 (52.3 %)	1.00	0.590
		GA	11 (55 %)	21 (47.7 %)	1.34 (0.46–3.87)	
	Log-additive	–	–	–	1.25 (0.59–2.68)	0.560

*p* ≤ 0.050 is considered significant

^a^ *n* number of examined fetuses and newborns

^b^ *OR* odds ratio

^c^ *CI* confidence interval

^d^Logistic regression model

### The prevalence rates of the alleles located at the *TLR4* and *TLR9* SNPs

In the *T. gondii*-infected fetuses and newborns, the prevalence rates of A and G alleles at the *TLR4* 896 A>G SNP were 97.4 % (37/38) and 2.6 % (1/38), respectively (see Table 3). Considering the *TLR4* 1196 C>T SNP, the prevalence rates of C and T alleles were 97.2 % (35/36) and 2.8 % (1/36), respectively. At the region of the *TLR9* SNP, the G allele was observed with a prevalence rate of 47.5 % (19/40), while the A allele was observed with a prevalence rate of 52.5 % (21/40). Among the uninfected control fetuses and newborns, within the *TLR4* 896 A>G polymorphic region, the A allele was observed with a prevalence rate of 95.0 % (95/100), while the G allele was observed with a prevalence rate of 5.0 % (5/100). At the *TLR4* 1196 C>T SNP, the C allele occurred with a prevalence rate of 91.5 % (75/82), while the T allele occurred with a prevalence rate of 8.5 % (7/82). In case of the *TLR9* SNP, the G allele was observed with a prevalence rate of 58.0 % (51/88), while the G allele was observed with a prevalence rate of 42.0 % (37/88). In case of the *TLR4* 1196 C>T SNP, the prevalence rate of the major allele was slightly increased among the infected patients compared to the uninfected controls. Taking into account all the analyzed alleles, the differences in their prevalence rates between the studied patient groups were not significant.

**Table 3:** Prevalence rates of the alleles at the *TLR4* and *TLR9* polymorphic sites

Gene polymorphism		No.^a^ of carriers with *TLR* alleles (%)		*p*-Value^b^
		Congenital toxoplasmosis	Uninfected control	
*TLR4* 896 A>G				
Alleles	A	37 (97.4)	95 (95.0)	0.542
	G	1 (2.6)	5 (5.0)	
*TLR4* 1196 C>T				
Alleles	C	35 (97.2)	75 (91.5)	0.252
	T	1 (2.8)	7 (8.5)	
*TLR9* 1635 G>A				
Alleles	G	19 (47.5)	51 (58.0)	0.271
	A	21 (52.5)	37 (42.0)	

*p* ≤ 0.050 is considered significant

^a^ *No.* number

^b^Pearson’s Chi-squared test

## Discussion

The multiple SNP analysis, performed in our study for both the *TLR4* and *TLR9* SNPs, showed the GTG variants at the *TLR4* 896 A>G, 1196 C>T, and *TLR9* 1635 G>A polymorphisms to be significantly less frequent among the fetuses and newborns with congenital toxoplasmosis as compared to the uninfected offspring. Previously, no study had ever shown any simultaneous contribution of *TLR4* and *TLR9* SNPs to the occurrence of *T. gondii* infection. However, a combined involvement of other TLRs was reported in the development of *T. gondii* infection in mice as well as in humans [25, 26]. Some participation of both TLR2 and TLR4 in triggering an immune response after *T. gondii* infection was observed in human macrophages, while a sort of cooperation among TLR7 TLR9, and TLR11 was shown in mice [8, 25, 26]. The outcomes of our study may suggest a contribution of all the analyzed polymorphic sites in the immune response against *T. gondii*. The studied *TLR4* SNPs were in linkage disequilibrium; however, no difference was observed in the distribution of *TLR4* haplotypes between the *T. gondii*-infected fetuses and newborns and the uninfected controls. Besides the lack of papers on the role of genetic changes within *TLR4* in the development of toxoplasmosis, *T. gondii* infection was reported to trigger *TLR4* to activate proinflammatory transcription factors and the expression of the pro-cytokines encoding genes [6, 8, 10, 27]. Considering the polymorphic variability of *TLR4*, some studies showed a certain functional association of *TLR4* SNPs with its activity [28–30]. The *TLR4* 896 A>G and 1196 C>T SNPs were described to result in co-segregating Asp299Gly and Thr399Ile missense mutations, respectively, that are localized in the ectocellular domain of the encoded protein [31, 32]. The mutations were reported to diminish the *TLR4* response to its ligand lipopolysaccharide (LPS) in humans, while the Asp299Gly modification was involved in the response interruption [31]. Additionally, the alteration caused a disruption of the *TLR4* α-helical protein structure and some extension of the β-strand [33]. The minor allele at the 896 A>G SNP was reported to influence the dimerization of *TLR4*, although its expression was not altered [28]. Another study also showed both *TLR4* 896 A>G and 1196 C>T SNPs to impair the TLR4/MD dimerization required for activation of the downstream signaling [29]. In our study, the prevalence of minor alleles at both *TLR4* SNPs was lower in congenitally infected fetuses and newborns, although the difference was not significant. The significantly lower frequency of GTG variants in the *T. gondii*-infected offspring might suggest a protective role of the mutations in the *TLR4* gene against the development of parasitic infection. Some previous studies showed a dual function of TLR4 in the immune response after *T. gondii* infection [34–37]. In addition to triggering TLR4 signaling, associated with activation of the immune response, the parasite was also reported to use this molecule to escape the immune responses [38–40]. Hence, the modified *TLR4* protein could be helpful for the host immune system to interrupt *T. gondii* dissemination in the infected organism. However, the number of fetuses and newborns congenitally infected with *T. gondii* and analyzed in the reported study was rather small; hence, the current results should be considered as preliminary and their further confirmation would require the analysis of a larger group of infected fetuses and newborns.

In the uninfected fetuses and newborns, the disruption of the TLR9 signaling pathway, engaged in the induction of type I IFNs, might also be driven by the altered TLR4 molecule. TLR4 was reported to be able to enter endosomes and to utilize TRAM and TRIF adapters to activate the IRF3 transcription factor that contributes to the production of type I IFNs [10, 41]. In turn, TLR9 was observed to cooperate with IRF3 to induce inflammatory responses in liposome-DNA-injected mice [42]. Similarly to TLR4, the TLR9 molecule was suggested to be utilized by *T. gondii* in an immunosuppressive activity of the parasite that results in the inhibited mobilization of intracellular TNF-α to the surface of murine neutrophils [43]. Regarding *TLR9* polymorphisms, it was reported that the C allele at the *TLR9* 1635 G>A SNP correlated with toxoplasmic retinochoroiditis in Brazilian children with ocular toxoplasmosis [14]. Since the 1635 G>A SNP at the *TLR9* gene causes a synonymous amino acid change but no alterations in the regulatory site, it was suggested that other polymorphisms might affect the immune response against *T. gondii* as well [14].

Considering the outcomes of our study, the protective role of the analyzed *TLR4* and *TLR9* SNPs against congenital infection with *T. gondii* seems to be fairly plausible. The GTG multiple variants at the *TLR4* and *TLR9* SNPs were significantly less frequent among the fetuses and newborns with congenital toxoplasmosis than in the uninfected controls. The contribution of the presented multiple variants to the immune response against *T. gondii* seems to be possible. However, we suggest further studies with larger groups of patients to confirm the role of *TLR* SNPs in congenital toxoplasmosis, as well as to investigate the mechanisms which drive the participation of *TLR* polymorphisms in the immune response against the parasite.
